# Poly(A)-binding proteins are required for diverse biological processes in metazoans

**DOI:** 10.1042/BST20140111

**Published:** 2014-08-11

**Authors:** Richard W.P. Smith, Tajekesa K.P. Blee, Nicola K. Gray

**Affiliations:** * MRC Centre for Reproductive Health, Queen's Medical Research Institute, University of Edinburgh, 47 Little France Crescent, Edinburgh EH16 4TJ, Scotland, U.K.

**Keywords:** development, gametogenesis, mRNA stability and mRNA localization, mRNA translation, neuron, poly(A)-binding protein (PABP)

## Abstract

PABPs [poly(A)-binding proteins] bind to the poly(A) tail of eukaryotic mRNAs and are conserved in species ranging from yeast to human. The prototypical cytoplasmic member, PABP1, is a multifunctional RNA-binding protein with roles in global and mRNA-specific translation and stability, consistent with a function as a central regulator of mRNA fate in the cytoplasm. More limited insight into the molecular functions of other family members is available. However, the consequences of disrupting PABP function in whole organisms is less clear, particularly in vertebrates, and even more so in mammals. In the present review, we discuss current and emerging knowledge with respect to the functions of PABP family members in whole animal studies which, although incomplete, already underlines their biological importance and highlights the need for further intensive research in this area.

## Introduction

**Figure 1:**
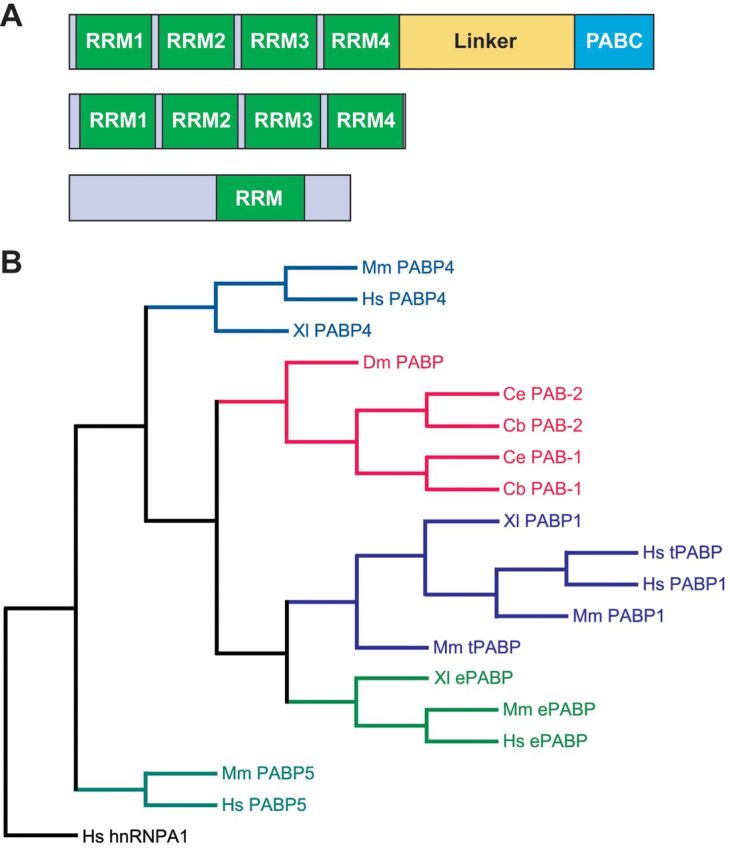
Relatedness and domain organization of PABP family members (**A**) Domain organization of PABPs. Top: vertebrate PABPC1, PABPC4, ePABP, tPABP (mammalian-specific), *D. melanogaster* dPABP and *C. elegans*/*C. briggsae* PAB-1 and PAB-2 (all predominately cytoplasmic). Middle: mammalian-specific PABPC5 (cytoplasmic). Bottom: PABPN1 (nuclear) and ePABP2 (cytoplasmic). Linker, proline/glutamine-rich variable linker region. (**B**) Phylogenetic tree of PABPC family proteins, rooted to human hnRNPA1 (heterogeneous nuclear ribonucleoprotein A1), an RRM-containing RNA-binding protein. Cb, *C. briggsae*; Ce, *C. elegans*; Dm, *D. melanogaster*; Hs, *Homo sapiens*; Mm, *Mus musculus*; Xl, *X. laevis*.

**Figure 2:**
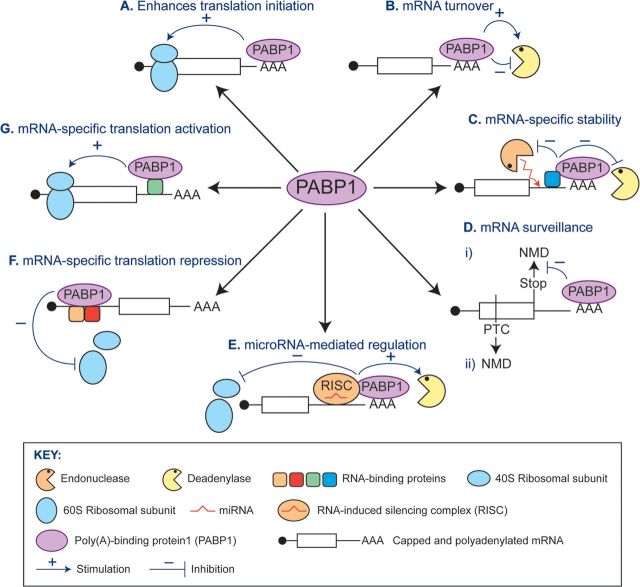
Molecular functions associated with PABP1 (**A**) PABP1 enhances global translation by binding to the poly(A) tail and interacting with factors at the mRNA 5′-end to recruit ribosomal subunits. (**B**) PABP1 stabilizes mRNAs by blocking access of deadenylases to the poly(A) tail, but also recruits deadenylases to the mRNA. This may be linked to translational termination. (**C**) An example of an mRNA-specific role in stability where PABP1 interaction with 3′-UTR-binding proteins blocks deadenylation and endonucleotic cleavage within the 3′-UTR. (**D**, panel i) PABP1 plays a role in correct termination at stop codons ensuring that the nonsense-mediated decay pathway is not activated. (**D**, panel ii) PABP1 does not participate in termination at premature termination codons (PTCs), termination is aberrant and mRNA decay ensues. (**E**) PABP1 enhances miRNA-mediated translational repression and deadenylation via its interaction with the miRNA-containing RISC complex. (**F**) An example of PABP1 acting in mRNA-specific translational repression when bound as part of a 5′-UTR repressive complex that blocks ribosome assembly. (**G**) PABP1 can act as an mRNA-specific activator when recruited to the 3′-UTR by other RNA-binding proteins or regulatory elements. Table 1 summarizes functions shown for different family members in the species discussed in this Figure.

All aspects of life require tightly regulated gene expression and recent studies have highlighted both the complexity and importance of post-transcriptional control mechanisms. RNA-binding proteins play a key role in exerting and co-ordinating such regulation. One class of RNA-binding proteins that regulate numerous aspects of eukaryotic mRNA fate comprises the PABPs [poly(A)-binding proteins]. Both PABPNs (nuclear PABPs) and PABPCs (cytoplasmic PABPs) bind the poly(A) tail, but consist of very distinct domains (Figure 1A) and have different steady-state intracellular distributions and functions [1–3]. The present review discusses metazoan PABPCs (referred to hereafter as PABPs), which vary in number between organisms (Figure 1B). PABP1, the prototypical member, has four N-terminal RRMs (RNA-recognition motifs), a linker region and a highly conserved PABC (PABP C-terminal) domain (Figure 1A). Where studied, PABPs show a predominantly diffuse cytoplasmic distribution [4,5], but can be enriched at sites of localized translation, e.g. neuronal dendrites [6] or leading edges of migrating fibroblasts [7]. At least some family members shuttle to and from the nucleus [4,8] and can, during cell stress, accumulate in the nucleus [5,8] or in cytoplasmic foci, e.g. stress granules [5,9]. Studies of the molecular functions of PABPs have mainly focused on PABP1 (also called PABPC1) and have mostly used reporter mRNAs either in mammalian cell-free extracts/cell lines or in *Xenopus laevis* oocytes. This has revealed multiple functions that are reviewed in [1–3,10,11] (Figure 2). Briefly, the best characterized function of poly(A)-tail-bound PABP1 is enhancing translation initiation by interacting with translation initiation factors bound at the 5′-end of the mRNA (Figure 2A): this is proposed to stabilize their interaction with mRNA, thereby enhancing the recruitment of ribosomal subunits. Although considered a ‘global’ effect, the extent to which translation of individual mRNAs is stimulated can be influenced by regulation of their poly(A)-tail lengths. When bound (directly or indirectly) to sites other than the poly(A) tail, PABP1 can also activate or repress translation in an ‘mRNA-specific’ manner (Figures 2F and 2G) depending on the location of its alternative binding sites and the proteins with which it interacts [10].

PABP1 also has multiple less well-characterized roles in mRNA turnover [11,12]. Of these, its best known role is protecting the poly(A) tail from deadenylation [poly(A) removal], the first and rate-limiting step in mRNA turnover (Figure 2B). Paradoxically, PABP1 also recruits deadenylases to mRNAs (Figure 2B) and has been suggested to co-ordinate translational termination with deadenylation, thereby regulating mRNA lifespan. Similar to translation, PABP1 has mRNA-specific roles in regulating mRNA stability (Figure 2C), either as part of regulatory complexes bound to sites within mRNAs or by interacting with stabilizing or destabilizing complexes when bound to the poly(A) tail. PABP1 is also involved in miRNA-mediated translational repression and/or deadenylation (Figure 2E) and in discriminating mRNAs which should undergo nonsense-mediated decay due to the presence of premature stop codons [3,11] (Figure 2D). Unlike their *Saccharomyces cerevisae* counterpart, mammalian PABPs do not participate in mRNA export [5].

The molecular functions of the other PABP family members are less well-characterized, but those of two vertebrate-specific PABPs (Figure 1), ePABP (embryonic PABP, also known as ePAB and PABPC1L) and PABP4 (also known as iPABP and PABPC4) have been examined in *X. laevis* egg extracts and oocytes (Table 1). Germ cells and early embryos undergo periods of transcriptional quiescence and are therefore heavily reliant on changes in mRNA translation which are often associated with dynamic poly(A)-tail length regulation in the cytoplasm: shortening (deadenylation) and extension (polyadenylation) lead to translational repression and activation respectively [13]. Cytoplasmic polyadenylation facilitates PABP recruitment and occurs in multiple cell types across metazoans (e.g. oocytes, male germ cells, early embryos and neurons [13]). All three *X. laevis* PABPs can stimulate poly(A)-dependent and mRNA-specific translation [14–16], both PABP1 and ePABP protect mRNAs from ‘default’ deadenylation [17,18] and ePABP can enhance cytoplasmic polyadenylation [19] and retard deadenylation driven by AREs (AU-rich elements) [18]. Although the ability of *X. laevis* PABP4 to regulate deadenylation has not been studied, mammalian PABP4 shows preference for AU-rich sequences in addition to poly(A) [20] and enhances translation and stability of ARE-containing mRNAs in extracts and/or cell lines [21,22]. Little is known about the functions of the two mammalian-specific PABPs, tPABP (testis-specific PABP, also known as PABPC2 and PABPC3) and PABP5 (PABPC5), which lacks the linker region and the PABC domain (Figure 1A). Finally, the cytoplasmic ePABP2 (or PABPN1-like) protein is outside the scope of the present review because it resembles PABPN [23,24] (Figure 1A).

**Table 1:** Molecular functions of PABPs Functions are shown in italics where direct evidence is absent and in bold where substantive or multiple lines of evidence are available. Indirect or contradictory experimental evidence is shown in italics. Protein–protein interactions and RNA binding have not been included for brevity. PABPs are only listed where evidence is available.

(a) Invertebrates		
PABP	Molecular function	Evidence
*D. melanogaster*	**Enhances translation (global and/or mRNA-specific)**	• *Polysome association* [72]
dPABP		• Depletion or depletion/add-back experiments with reporter mRNAs [39,73,74]
	**miRNA-mediated regulation**	• Depletion/add-back experiments with reporter mRNAs [74]
		• Reporter assays using mutants that impede dPABP protein interactions [75,76]
		• Overexpression experiments using tethering assays [75]
	**mRNA surveillance**	• Reporter and tethering assays [77]
		• Knockdown effects on reporter and endogenous mRNA [77]
	*mRNA-specific stability*	• *Effect of pAbp hypomorphs on mRNA absundance* [34]
*C. elegans* PAB-1	*Enhances translation initiation*	• *Polysome association* [78]
(b) Non-mammalian vertebrates		
PABP	Molecular function	Evidence
*X. laevis*		
PABP1	**Enhances translation (global and/or mRNA-specific)**	• Tethering assays [14]• Knockdown studies with metabolic labelling [15]
	**Inhibits deadenylation**	• Overexpression studies [17]
ePABP	**Enhances translation (global and/or mRNA-specific)**	• *Polysome association* [16]• Tethering assays [16,19]• Knockdown studies with metabolic labelling [14–16]
	**Cytoplasmic polyadenylation**	• Sequestration/add-back with endogenous mRNAs [19]
	**Inhibits deadenylation**	• Depletion effects on reporter mRNAs [18,79]
PABP4	**Enhances translation (global and/or mRNA-specific)**	• *Polysome association* [15]• Tethering assays [15]• Loss of polysomes following knockdown [15]
(c) Mammals		
PABP	Molecular function	Evidence
PABP1	**Enhances translation (global and/or mRNA-specific)**	Extensively reviewed [1–3,10]
	**mRNA turnover/mRNA-specific stability**	Recently reviewed [11]
	**mRNA surveillance**	Recently reviewed [3,11,80]
	**miRNA-mediated regulation**	Recently reviewed [3,75]
	**mRNA-specific translation repression**	Recently reviewed [10,81]
ePABP	**Cytoplasmic polyadenylation**	• Effect of knockout on endogenous mRNAs [64]
tPABP	*Enhances translation (global and/or mRNA-specific)*	• *Slightly augments reporter mRNA translation in vitro, but contradicted by lack of polysome association* [69]
PABP4	Enhances translation (global and/or	• Slight augmentation of reporter mRNA translation [21]
	mRNA-specific)	• *Polysome association* [82]
	**mRNA-specific stability**	• Effect of knockdown on endogenous mRNAs [22]

In the present review, we describe our current state of knowledge regarding PABP function in whole organism studies in animals, but do not address studies of plants, yeast, viral infection or parasites [3,12,25–28].

## Invertebrates

### Drosophila melanogaster

*D. melanogaster* encodes one PABP1 homologue, dPABP (or PABP55B) whose molecular functions are incompletely characterized (Table 1), but which appears essential for viability since compound heterozygous deletions have an embryonic lethal phenotype at an unspecified stage [29]. Other studies, mostly using different P-element (transposon) insertions at the *pAbp* locus [29–34], have identified a range of phenotypes (described below). Although classified as hypomorphs (i.e. reduced gene activity or function), information concerning their actual effect on PABP function/expression (all but one lie outside the *pAbp* ORF) is lacking unless stated otherwise. Therefore the extent of dPABP insufficiency associated with these phenotypes remains unclear. Similarly, although some studies have used multiple mutant alleles, transgene rescue experiments to eliminate off-target effects are lacking, unless indicated otherwise.

Nonetheless, several studies suggest dPABP is important within the germline and following fertilization. *D. melanogaster* developing oocytes are supported by nurse cells that provide nutrients, RNAs and proteins. Localized translation of mRNAs [e.g. *bcd* (*bicoid*), *osk* (*oskar*), *nos* (*nanos*) and *grk* (*gurken*)] following transport from the nurse cells to particular sites within the oocyte, results in protein gradients that establish embryonic body axes and the germline. Correct spatiotemporal translation of these mRNAs requires multi-protein complexes to co-ordinate their localization, translational repression and activation [35].

Homozygous or compound heterozygous *pAbp* mutations, one of which was rescued by a dPABP transgene [34], results in arrest of oogenesis at stage 3 [31], or stages 5–6 (of 14 stages) [34], demonstrating an early requirement for dPABP during this process. Further analysis revealed that oocyte growth and positioning and egg chamber packaging were affected as mutant egg chambers contained abnormally small mis-positioned or multiple oocytes. Nurse and follicle cell development was also affected.

Some insight into dPABP function in these early oocytes comes from its association and co-localization with a protein complex that is involved in the microtubule-mediated transport of *osk*, *bcd* and *grk* mRNAs from nurse cells to the oocyte [35]. This association may be indirect as it is RNA-dependent, but studies in oocytes containing different compound heterozygous *pAbp* mutations show that dPABP is essential for posterior accumulation of *osk* [but not *grk* and *bic* (*bicaudal*)*-D*] mRNA [34] and for the localization of Staufen protein, which is interdependent with that of *osk* mRNA [36]. The specificity of dPABP for *osk* mRNA may be due to the presence of adenine-rich tracts within the *osk* mRNA 3′-UTR which bind dPABP *in vitro* [34].

Although a direct role for dPABP in the mRNA localization process cannot be ruled out, the failure to accumulate localized *osk* mRNA may be attributable to a role of dPABP in maintaining *osk* mRNA stability, since *osk* mRNA abundance is reduced in oocytes with different compound *pAbp*-heterozygous mutant alleles [34]. This stabilizing function may also explain why dPABP deficiency causes *osk* haploinsufficiency and why reducing *pAbp* to one copy suppresses patterning defects caused by anterior mis-localization of *osk* mRNA in *bic-D* mutants [34].

Subsequent to this early role, dPABP appears to be required for the spatiotemporal control of other mRNAs that establish embryonic protein gradients, e.g. via localized Grk synthesis. Eggs from several homozygous and compound heterozygous *pAbp* hypomorphs display patterning defects suggestive of reduced dorso-anterior Grk protein levels [31], and heterozygous *pAbp* hypomorphs exacerbate patterning defects in *grk* mutants [31]. Interestingly, dPABP can be co-isolated with Cup and Encore (Enc), proteins that function in *grk* mRNA localization and either its translational repression or activation respectively [31,37]. *pAbp* and *cup* mutants lay eggs with opposite patterning defects (‘ventralized’ compared with ‘dorsalized’ respectively), whereas a heterozygous *pAbp* hypomorph enhances the ventralized and collapsed egg phenotypes of *enc* mutants [31]. This suggests that dPABP may antagonize Cup-mediated repression and, similar to Enc, is required to promote *grk* mRNA translation.

dPABP may also contribute to translational repression of *cad* (*caudal*) mRNA [33] by a Bcd-containing complex which establishes the posterior–anterior gradient of Caudal [38]. Bin-3 (bicoid-interacting protein 3) is also implicated in this repression and the levels of embryonic lethality of a *bin3 bcd* double mutant are enhanced by a *pAbp* heterozygous hypomorph [33], but it is not clear whether dPABP contribution to *cad* mRNA repression is direct.

A later role in determining body morphology is suggested by studies of wing size. Heterozygous *pAbp* hypomorph flies or those overexpressing an inhibitor of translation that sequesters dPABP, called dPaip2 (*D. melanogaster* PABP-interacting protein 2), in wing-imaginal discs have reduced wing size [39]. Importantly, co-overexpression of dPABP with dPaip2 rescues wing cell number and size [39].

In addition to affecting oogenesis and body pattern, dPABP is also required for spermatogenesis [30,32] as several compound heterozygous *pAbp* mutant flies are male sterile and display aberrant meiosis (only undergoing one meiotic division), spermatid elongation and/or cytokinesis [30,32]. Although the molecular events leading to these defects are unknown, the *pAbp* meiotic phenotype partially overlaps with that of Larp (La-related protein), a factor thought to regulate mRNA stability in *Caenorhabditis elegans* and translation of certain mRNAs in mammalian cells [40–42], which interacts biochemically and genetically with dPABP [30].

Many mRNA-binding proteins that function in the germline also appear to be important in neurons. Neurons pass signals to other cells via synapses and localized translation at these sites is thought to regulate synaptic plasticity [43]. Aggregates containing dPABP and eIF4E (eukaryotic initiation factor 4E) coincide with polyribosome clusters within subsynaptic compartments of larval NMJs (neuromuscular junctions), posited sites of localized translation [29]. Mutants that increase synaptic activity, or larvae overexpressing *pAbp* mRNA show increased occurrence of subsynaptic dPABP/eIF4E aggregates, altered levels of some synaptic proteins, significantly larger NMJs and more efficient neurotransmission [29], consistent with modified subsynaptic translation. However, similar observations were made with reduced *pAbp* mRNA levels (heterozygous *pAbp* hypomorph) and neither increased nor decreased *pAbp* mRNA levels were reflected in dPABP protein levels [29], making the observed alterations in dPABP/eIF4E aggregates and synaptic activity difficult to explain.

Studies of dFMR1, the *D. melanogaster* homologue of FMRP (fragile-X mental retardation protein), also link dPABP to synaptic plasticity. FMRP is an RNA-binding protein that regulates mRNA localization, translation and stability [44] and is important for cognition. dFMR1 is required for long-term memory [45] and a genetic screen for genes involved in dFMR1-mediated translational repression identified *pAbp* [46]. dPABP co-localizes with dFMR1-positive neuritic RNPs (ribonucleoproteins) and its overexpression inhibits dendritic branching, suggestive of a function in translational repression [46], but this activity awaits confirmation.

Interestingly, the phenotypes of several *D. melanogaster* models of human neurodegenerative diseases are also affected by dPABP. For instance, neurodegeneration in SCA3 (spinal cerebellar ataxia 3) models is exacerbated by heterozygous *pAbp* deletion and reduced by dPABP overexpression [47]. Similarly, PABP1 accumulates in cytoplasmic inclusions in motor neurons from ALS (amyotrophic lateral sclerosis) patients [48] and siRNA-mediated dPABP knockdown in fly models of ALS suggests it is required for inclusion formation [48].

### C. elegans and Caenorhabditis briggsae

*C. elegans* and *C. briggsae* encode two PABPCs, PAB-1 and PAB-2 ([49]; WormBase) whose molecular functions largely await characterization (Table 1). In *C. elegans*, individual *pab-1* (detailed in [50]) or *pab-2* knockdown leads to limited somatic defects such as abnormally protruding vulva (high and low penetrance respectively; also seen in a *pab-1* nonsense mutation), low penetrance ruptured vulva, and flaccid body morphology (*pab-1* only) [49,51].

In both species, *pab-2* is X-chromosomal. Consistent with germline X-chromosome inactivation, its knockdown does not affect *C. elegans* fertility [49]. However, despite conservation of chromosome silencing [52], knockdown in *C. briggsae* drastically affects embryo number and mortality [49], which is suggestive of reproductive and/or developmental defects.

In contrast, *pab-1* is essential for fertility in both species [49,51,53–56]. *C. elegans* is a hermaphrodite, which first makes sperm and then switches to oogenesis, and knockdown at different stages of post-embryonic development revealed PAB-1 is required throughout gametogenesis. Even late-stage PAB-1 depletion is deleterious, leading to defective oogenesis, high embryonic death and infertility of surviving progeny [54]. This suggests that PAB-1 is likely to regulate mRNAs that function at different stages of germline development [54]. PAB-1 is present in, and reportedly required for the formation of, P-granules [54], germ-cell specific cytoplasmic foci, which are considered centres of post-transcriptional regulation as they are rich in RNA-binding proteins and mRNA [57]. Although P-granules are implicated in both germline proliferation and gametogenesis, it is unclear whether the association of PAB-1 with these foci accounts for its essential germline function.

Germ cell ablation can lead to longevity and gigantism [58,59] and *pab-1* nonsense alleles increase lifespan 1.3–1.5-fold [49,53] and body volume 1.4-fold [49], which may be a consequence of the described germline defects. Curiously, *pab-2* deletion alters relative lifespan when worms are fed on different bacterial species [60].

## Vertebrate phenotypes associated with loss of PABP function

### X. laevis

*X. laevis* PABPs exhibit distinct distributions: in adult tissues, PABP1 is widely expressed albeit at variable levels [16,61], and PABP4 mRNA is widely distributed [15], whereas ePABP is restricted to the gonads [16,61]. In oocytes and early embryos, ePABP is the predominant PABP [15,16,18]. Changes in ePABP phosphorylation during oocyte maturation, when fully grown oocytes become fertilization competent, suggest regulation of its activity coincident with changes in the poly(A)-tail length and translation of many mRNAs [19]. Oocyte maturation is impeded by ectopic xPAIP2 (*X. laevis* PABP-interacting protein 2)-mediated PABP sequestration which blocks the cytoplasmic polyadenylation of mRNAs whose translational activation is required for maturation. Rescue by overexpression of ePABP, but not a form in which several phospho-residues have been mutated, demonstrates the importance of ePABP and these phosphorylations for oocyte maturation [19].

Each PABP is essential for *X. laevis* embryonic development and viability [15]. Morpholino-mediated knockdown of PABP1 leads to a range of morphological phenotypes in tadpoles (e.g. abnormal development of the eye, cement gland, tail and fin, and body curvature), problems with movement and embryonic death by stage 30/31 (out of 66 developmental stages) [15]. ePABP knockdown results in similar morphological and movement defects, but, surprisingly, death occurs later, by stage 35, perhaps due to the higher levels of ePABP in early embryos delaying effective knockdown [15]. In contrast, PABP4 knockdown results mainly in anterior morphological defects (e.g. cephalic and ventral oedema, malformation of the head, poor eye development, and digestive tract deformities) and abnormal swimming motions. PABP4 phenotypes become apparent later than those of PABP1 and ePABP- and PABP4-deficient embryos do not die until stage 50 [15]. Importantly, the respective phenotypes were recapitulated with multiple morpholinos and could be effectively rescued [15].

For each of these knockdowns, the developmental defects were accompanied by significant decreases in global protein synthesis, suggesting a potential molecular basis for the phenotypes [15]. However, despite this commonality, cross-rescue experiments showed that neither ePABP nor PABP4 could fully rescue PABP1 knockdown, indicating functional differences must also exist between individual PABP family members, which domain-swap experiments showed to be conferred by multiple domains [15]. The ability of all three PABPs to stimulate global translation [15] (Table 1) suggests that their non-redundant functions may relate to individual roles in regulating mRNA-specific translation or mRNA decay.

### Mouse

Insight into the phenotypic consequences of loss of PABP function in mammals is only available for Epab. Murine *Epab* mRNA is only present in male and female germ cells and one- and two-cell stage embryos [62,63]. In contrast with ePABP-deficient *X. laevis*, *Epab*^−/−^ mice display no growth or developmental abnormalities, a difference that may reflect reduced reliance on post-transcriptional regulation in mouse embryos since zygotic transcription begins at the two-cell stage in mice rather than at mid-blastula transition in *X. laevis*.

*Epab*^−/−^ females, but not males, are sterile [64,65]. In mammals, hormonal signals trigger the development and maturation of oocytes within follicles that progress through a series of developmental stages and contain somatic cells that respond to and support the growing oocyte. *Epab*^−/−^ mice have normal oestrus and normal follicle numbers at all stages, with the exception of secondary follicles which are overly abundant, but their oocytes fail to mature either *in vivo* or *in vitro*. Similar to what was described previously for *X. laevis* [19], this maturation defect appears to be due, at least in part, to abrogated cytoplasmic polyadenylation, resulting in reduced expression of proteins required for oocyte maturation [64]. However, ePABP may also be required earlier in oogenesis, since injection of *Epab* mRNA into fully grown *Epab*^−/−^ oocytes failed to rescue maturation *in vitro* [64].

Following ovulation, follicles become corpora lutea which, in superovulated *Epab*^−/−^ mice, show increased retention of oocytes, indicative of defective ovulation [64]. This phenotype appears to result from somatic cell defects in the follicle which are likely indirect as these cells do not express *Epab* [64].

## Cell-based models of red blood cell maturation

In a recent study, a potential role for mammalian PABP4 in red blood cell maturation was identified [22]. Mature red blood cells lack a nucleus making their terminal differentiation highly dependent on post-transcriptional control; this can be modelled by dimethylsulfoxide treatment of MEL (lymphoma-derived murine erythroleukaemia) cells. Intriguingly, shRNA-mediated depletion of PABP4 in MEL cells increased or decreased the abundance of limited mRNA subsets and hindered terminal MEL cell differentiation [22]. Although the underlying mechanism requires clarification, it was suggested that AREs in some of these mRNAs may aid PABP4 binding to impede their rapid decay following deadenylation.

## Perspectives

Although recent work has provided fascinating insight into the complexity of the biological processes in which PABP family members are involved, their molecular multi-functionality suggests that we have only scratched the surface. Indeed, even in invertebrates where PABPs are already implicated in diverse phenotypes, they may be involved in additional processes, e.g. circadian rhythm and transposon silencing [66–68]. Our knowledge is more limited in vertebrates, which encode a greater diversity of PABPs, with only their roles in early development and oogenesis having been explored. In mammals, the critical role of ePABP in oocytes is conserved, but information on other PABPs is not available although extrapolating from non-mammalian studies suggests that mammalian PABP1 may be essential. It remains to be determined whether mammalian PABP4 plays an analogous role to its *X. laevis* counterpart in development and whether its effects on mammalian erythroid differentiation are recapitulated *in vivo*. There is no insight into the roles of tPABP and PABP5 from cell lines or other models, but tPABP is only expressed in a subset of male germ cells, indicating its function is restricted to the male germline [69], where it may be redundant with PABP1. Consistent with this idea, tPABP interacts with translation factors and can stimulate reporter mRNA translation *in vitro* [69] (Table 1). However, tPABP appears not to be polysome-associated and discrepancy exists as to whether its distribution within cytoplasmic foci called chromatoid bodies is distinct from that of PABP1 [69,70]. Little is known about the expression of PABP5, whose domain structure (Figure 1A) suggests distinct roles from other PABPs, although intriguingly, a truncated PABP5 isoform is present in mitochondria, suggesting a potential function in these organelles [71]. In conclusion, further investigation into the roles of PABPs in whole organisms, complementing molecular studies that underscore their central role in cytoplasmic mRNA metabolism, should uncover the full extent of their importance in both normal and diseased states.

## Abbreviations

ARE: AU-rich element
Bcd: Bicoid
Bic-D: Bicaudal-D
Bin: Bicoid-interacting protein
Cad: Caudal
dFMR1: *D. melanogaster* FMRP1
dPABP: *D. melanogaster* PABP
dPaip2: *D. melanogaster* PABP-interacting protein 2
eIF: eukaryotic initiation factor
Enc: Encore
ePABP: embryonic PABP
FMRP: fragile-X mental retardation protein
Grk: Gurken
MEL: lymphoma-derived murine erythroleukaemia
NMJ: neuromuscular junction
Osk: Oskar
PABC: PABP C-terminal
PABP: poly(A)-binding protein
PABPC: cytoplasmic PABP
PABPN: nuclear PABP
RRM: RNA-recognition motif
tPABP: testis-specific PABP
